# The Beneficial Effects of Bariatric-Surgery-Induced Weight Loss on Renal Function

**DOI:** 10.3390/metabo12100967

**Published:** 2022-10-12

**Authors:** Diego Moriconi, Monica Nannipieri, Prince Dadson, Javier Rosada, Nikolaos Tentolouris, Eleni Rebelos

**Affiliations:** 1 Department of Clinical and Experimental Medicine, University of Pisa, 56126 Pisa, Italy; 2 Turku PET Centre, University of Turku, 20500 Turku, Finland; 3 Fourth Unit of Internal Medicine, University Hospital of Pisa, 56124 Pisa, Italy; 4 Department of Propaedeutic and Internal Medicine, Medical School, National and Kapodistrian University of Athens, Laiko General Hospital, 11527 Athens, Greece; 5 Institute of Clinical Physiology, National Research Council (CNR), 56124 Pisa, Italy

**Keywords:** obesity, bariatric surgery, renal function, renal metabolism, renal perfusion, renal sinus fat

## Abstract

Obesity represents an independent risk factor for the development of chronic kidney disease (CKD), leading to specific histopathological alterations, known as obesity-related glomerulopathy. Bariatric surgery is the most effective means of inducing and maintaining sustained weight loss. Furthermore, in the context of bariatric-surgery-induced weight loss, a reduction in the proinflammatory state and an improvement in the adipokine profile occur, which may also contribute to the improvement of renal function following bariatric surgery. However, the assessment of renal function in the context of obesity and following marked weight loss is difficult, since the formulas adopted to estimate glomerular function use biomarkers whose production is dependent on muscle mass (creatinine) or adipose tissue mass and inflammation (cystatin-c). Thus, following bariatric surgery, the extent to which reductions in plasma concentrations reflect the actual improvement in renal function is not clear. Despite this limitation, the available literature suggests that in patients with hyperfiltration at baseline, GFR is reduced following bariatric surgery, whereas GFR is increased in patients with decreased GFR at baseline. These findings are also confirmed in the few studies that have used measured rather than estimated GFR. Albuminuria is also decreased following bariatric surgery. Moreover, bariatric surgery seems superior in achieving the remission of albuminuria and early CKD than the best medical treatment. In this article, we discuss the pathophysiology of renal complications in obesity, review the mechanisms through which weight loss induces improvements in renal function, and provide an overview of the renal outcomes following bariatric surgery.

## 1. Introduction

Bariatric surgery is the most effective means of inducing and maintaining sustained weight loss. Apart from the weight loss effect, bariatric surgery leads to the remission of chronic metabolic disorders such as type 2 diabetes (T2D) [1] and hypertension [2].

Both obesity and T2D represent independent risk factors for chronic kidney disease (CKD) [3,4]. Currently, more than 850 million people are affected by CKD worldwide, a condition linked to substantially increased mortality and impaired quality of life [5]. These numbers, in combination with the low awareness of CKD and the projection that renal replacement therapy will increase substantially in the next few decades, make CKD a major health problem.

In the present article, we review the literature regarding the pathophysiology leading to chronic kidney disease in the context of obesity and the effects of bariatric surgery on renal function, structure, metabolism, and perfusion. A special mention is made of the challenges in evaluating renal function following weight loss, since in this setting, the assessment of renal function with estimates based on creatinine or cystatin-c levels is biased due to the contemporaneous reduction in lean and fat mass, respectively [6].

## 2. Search Strategy and Selection Criteria

We searched PubMed and Google Scholar for articles published up to August 2022, using the search terms: “renal function”, “chronic kidney disease”, “GFR”, “glomerular filtration rate”, “albuminuria, “proteinuria”, “nephrolithiasis”, “renal metabolism”, “renal perfusion”, “renal sinus fat”, “obesity”, and “bariatric surgery”. We also searched the reference lists of the articles identified by this search strategy and selected relevant titles. We supplemented the search with records of relevant publications from our personal files.

### 2.1. Structural and Functional Renal Alterations Occurring in the Context of Obesity

Numerous cross-sectional and cohort studies have shown an association between obesity and both the presence and the development of chronic kidney disease (CKD) [7,8,9]. In large population-based studies, a higher BMI was associated with a more rapid decrease in estimated glomerular filtration rate (eGFR) over time [9] and with the incidence of end-stage kidney disease (ESKD) [10,11]. Furthermore, severe obesity was shown to be associated with a more rapid reduction in GFR in patients with pre-existing CKD [12]. A previous meta-analysis showed that overweight and obesity accounted for approximately for 13.8% and 24.9% of kidney disease, without sex differences, in industrialized countries [4].

Obesity is associated with the risk of developing kidney damage through both direct and indirect mechanisms, by favoring the development of T2D and hypertension [13]. In this respect, obesity per se causes various structural and functional renal alterations, likely as an adaptation to modified hemodynamic conditions. Previous autopsy studies have shown that patients with obesity have larger kidney diameters and heavier kidneys compared to normal-weight controls [14]. Furthermore, as demonstrated in more recent morphometric studies performed using kidney biopsies, obesity is associated with a higher glomerular size [15], even in the absence of overt kidney disease [16]. This phenomenon may be related to the compensatory hypertrophy of single nephrons following the increase in metabolic demands. In addition to the glomeruli, tubules are also affected. Kidney biopsies from non-diabetic obese patients with proteinuria have demonstrated that obesity-related glomerular modification is associated with proximal tubular epithelial hypertrophy and increased tubular urinary space compared to lean non-proteinuric controls [17].

As previously mentioned, parallel to structural changes, obesity is associated with functional modifications that reflect changes in hemodynamics. GFR and renal plasma flow (RPF) both increase with obesity, as well as the ratio between these two parameters, the so-called filtration fraction (RPF/GFR) [18]. The elevation in the filtration fraction leads to an increase in the reabsorption of sodium and water in the proximal convoluted tubules, resulting in decreased afferent arteriolar resistance via the tubuloglomerular feedback mechanism [19]. From this altered arteriolar balance derives the phenomenon of glomerular hyperfiltration, which can be considered the first step of subclinical damage in severe obesity. Once the mechanism of hyperfiltration is triggered, a series of histological modifications may occur, and these can lead to the transition from pre-clinical states to overt nephropathy, such as microalbuminuria, subnephrotic and nephrotic proteinuria, and a progressive reduction in GFR [20].

In the last few decades, the progress in the knowledge on the renal involvement in obesity has made it possible to identify a specific histopathological disease, known as obesity-related glomerulopathy (ORG) [20]. The histologic features of ORG include glomerulomegaly with reduced glomerular density and focal segmental glomerulosclerosis (FSGS) [21]. In ORG, although not always present, the pattern of FSGS consists of perihilar segmental sclerosis, associated in the majority of cases with peripheral sclerotic lesions that typically affect hypertrophied glomeruli. This specific histological picture may reflect a redistribution of the hemodynamic pressure load on the vascular pole of the glomeruli [21]. In the end, the percentage of glomeruli affected by segmental sclerosis tends to be lower in ORG than in primary FSGS, and this is consistent with the clinical course of ORG, which is typically less aggressive [22].

### 2.2. Glomerular Hyperfiltration in Severe Obesity Is the Trigger for the Development of ORG

Hyperfiltration is a phenomenon that was first postulated by Brenner et al. [23], who showed that unilateral nephrectomy caused proteinuria and sclerosis in the contralateral kidney in murine models. The theory of hyperfiltration is based on the hypothesis that a reduction in the number of healthy nephrons leads to global adaptation in the rest, which are forced to take over the function in order to keep the GFR stable. Thus, the residual glomeruli are characterized by an increase in glomerular capillary pressure and tensile stress, which may represent the first stage of subclinical kidney damage [24], potentially leading to podocyte loss and glomerulosclerosis [25]. However, although obesity in the absence of comorbidities is itself associated with hyperfiltration, the main mechanism involved is yet to be fully elucidated.

A prospective study involving 194 Pima Indians with different stages of insulin resistance demonstrated that the GFR was higher in patients with impaired glucose tolerance compared to their normoglycemic counterparts [26], while another study including predominantly overweight/obese normoglycemic individuals showed that, although measures of adiposity were positively related to GFR, the degree of insulin resistance measured by the euglycemic hyperinsulinaemic clamp was the most important parameter associated with hyperfiltration according to the multivariate analysis [27]. This suggests that insulin resistance may tip the balance in the development of glomerular hyperfiltration. It is known that chronic low-grade inflammation, which is triggered by the activation of the NLRP3 inflammasome, is the common denominator in insulin resistance and obesity [28], and it has been shown that serum levels of interleukin-1β correlate inversely with insulin sensitivity [29].

Furthermore, evidence suggests a link between endothelial dysfunction and obesity, especially in a condition associated with a proatherosclerotic phenotype in which the inflammatory burden is known to play a pivotal role [30].

On the basis of these published studies, the following pathophysiological steps can explain the link between obesity and hyperfiltration, the first step in renal disease. First, the disruption in adipose tissue function leads to a chronic inflammatory state and to the dysregulation of the endocrine actions of adipocyte-derived factors [31]. On the one hand, this favors the development of insulin resistance; on the other hand, it leads to endothelial dysfunction. Insulin resistance and endothelial dysfunction could be the key factors involved in the onset of hyperfiltration and in subsequent chronic renal damage in severe obesity.

In support of the importance of inflammation in inducing hyperfiltration, a recent study showed that in severe obesity, after bariatric surgery and the consequent impressive weight loss, the GFR remained abnormally high in about 30% of subjects who did not have a reduction in IL-1β and caspase-1, suggesting the pathogenetic role of inflammasome signaling in perpetuating hyperfiltration [32].

A key adipokine for the development of ORG is represented by leptin, which increases both in conditions of insulin resistance and through inflammatory mechanisms mediated by Il-1β and TNF-α [33,34], highlighting the link between inflammation and insulin resistance as pathogenetic factors not only of hyperfiltration, but also of the subsequent histological steps characterizing ORG. Leptin stimulates the expression of collagen type IV production, promoting an increase in the extracellular matrix and renal fibrosis [35] and the development of a tubular fibrogenic response via TGF-β [36]. Furthermore, the increased expression of IL-6 receptors and signal transducers has been shown in the glomeruli of individuals affected by ORG, suggesting the role of IL-6 in the progression of kidney disease [37].

Obesity, whether it is associated with hyperlipidemia or not, aggravates intracellular ectopic lipid accumulation [38]. In fact, the endothelial dysfunction increases lipoprotein leakage in the glomerular tuft, while the low-grade inflammatory state interferes with LDL receptor feedback, causing lipid accumulation in mesangial, endothelial, and tubular cells [39]. Lipotoxicity causes a decrease in podocyte number and density in ORG [38], and the damage of the glomerular filtration barrier leads to the development and worsening of proteinuria. Furthermore, intracellular lipid accumulation in cells rich in mitochondria such as those that make up the proximal tubules leads to mitochondrial dysfunction, mainly via excessive reactive oxygen species production [40], contributing to renal fibrosis [41].

A recent in vitro study showed that adiponectin ameliorated FFA-induced podocyte injury, downregulating the ROS/NLRP3 pathway [42]. These data suggest that the altered balance between adipokines such as adiponectin and leptin contributes to the pathogenesis of ORG.

### 2.3. The Difficulty in Evaluating GFR before and after Bariatric Surgery: The Limits of the GFR Estimation Formulas

In clinical practice, renal function is estimated from GFR (eGFR) using serum creatinine or, sometimes, serum cystatin-c levels. However, both of these renal function markers have the significant limitation that they are influenced by changes in muscle and fat mass. In fact, creatinine generation is directly related to free fatty mass, while cystatin-c can be affected by fat mass [6]. Since the amount of fat and lean mass in patients with obesity is greater compared to normal-weight subjects, and the commonly used eGFR equations, adjusted for body surface area (BSA), were created using older data from populations with a lower prevalence of obesity [43], there are systematic errors in the estimation of the actual renal function in severe obesity [44].

Specifically, eGFR formulas that included weight, height, or BSA had an error that increased with increasing BMI, and the adjustment for BSA (i.e., the assumed 1.73 m^2^ of BSA used in the adjusted eGFR formulas) led to a significant underestimation of the renal function, resulting in an overestimation of the severity of CKD and, at the same time, an underestimation of the first stage of kidney injury due to hyperfiltration [44].

On the contrary, formulas for the estimation of creatinine clearance, such as those of Cockcroft and Gault, and the eGFR formulas unadjusted for BSA (mL/min) (GFR adjusted = (GFR unadjusted/BSA) × 1.73) overestimate the real renal function, because in severe obesity there is a disproportionate amount of fat mass as a percentage of body weight compared to in normal-weight subjects, which does not contribute to creatinine production [45].

After bariatric surgery, creatinine production decreases by about 20–25%, due to the loss in not only fat mass, but also muscle mass, resulting in a decrease in serum creatinine levels [46]. Therefore, using the adjusted eGFR formulas, we usually find an increase in eGFR following bariatric surgery as result of a reduction in serum creatinine levels. The extent to which this is due to the positive effect per se of weight loss on renal function, and the extent to which this is due to the weight loss itself is therefore difficult to discern when using eGFR. Vice versa, with the noticeable reduction in BSA following weight loss, unadjusted eGFR formulas may underestimate the true eGFR variation, because fat mass is reduced to a greater extent than muscle mass.

Cystatin-c, although initially promising, proved to be an imperfect marker for assessing kidney function in severe obesity. Cystatin-c is associated with fat mass and inflammation, which in turn are related to conditions of severe obesity [47]. Thus, once again following significant weight loss, it is not clear whether changes in cystatin-c levels reflect changes in the generation of this marker (due to a reduction in fat mass or inflammatory burden) or indicate an effective improvement in GFR.

In summary, in renal function evaluation before and after bariatric surgery, there are two main concerns. First, the correct estimation of GFR in obese patients is complicated, and eGFR equations fail to reflect real renal function. Second, the drastic changes in body composition make the observed changes in eGFR difficult to interpret. These concerns can be overcome by actually measuring rather than estimating GFR, and by evaluating markers of kidney damage, such as albuminuria in longitudinal studies.

### 2.4. The Effect of Bariatric Surgery on Estimated GFR and Albuminuria

In a large study, Chang and colleagues investigated the impact of bariatric surgery on renal outcomes in 985 patients (baseline type 2 diabetes in 37.8%; Roux-en-Y gastric bypass (RYGB) 96.5%, vertical sleeve gastrectomy (SG) 3.5%) and an equal number of matched controls with a median of 4.4 and 3.8 years of follow up [48]. Bariatric surgery reduced the risk of ≥30% eGFR decline by 58% (hazard ratio 0.42; 95% CI 0.32–0.55) and was associated with a 57% lower risk of the doubling of serum creatinine or end-stage kidney disease (ESKD). The beneficial impact of bariatric surgery was similar among patients with higher or lower eGFR. Risk reductions of similar magnitudes were also reported in the SOS study, in which the proportion of patients who underwent RYGB was lower (13%), and there was a smaller prevalence of T2D (about 7%) [49].

The Longitudinal Assessment of Bariatric Surgery (LABS)-2 investigated the impact of bariatric surgery on the Kidney Disease: Improving Global Outcomes (KDIGO) CKD classification over up to 7 years of follow up. On the basis of the combination of eGFR and albuminuria, patients were divided into four classes of CKD risk: low risk (83%), moderate risk (12%), high risk (3.4%), and very high risk (1.4%). The prevalence of T2D was 28% in patients with a low CKD risk and reached 83% in those with a very high CKD risk. At the 7-year follow up, improvements were observed in 53% of patients with moderate baseline CKD risk and 56% of those with high baseline risk. Furthermore, 23% of those with very high baseline CKD risk improved their risk category after 7 years, demonstrating the renoprotective effects of bariatric surgery across the whole spectrum of CKD classes, although less pronounced in the very-high-risk CKD category. The proportion of patients whose CKD risk worsened was ≤10%, and only five patients developed ESKD. Sensitivity analyses using year 1 as baseline to minimize the effect of weight loss on serum levels of creatinine, and differing eGFR formulas, yielded similar results [50].

In a 16-year retrospective study of individuals who underwent bariatric surgery, Romero-Funes et al. studied changes in eGFR, albuminuria, and kidney failure risk before, 3 months after, and one year after bariatric surgery (SG 61%, RYGB 39%). After the one-year follow up, in 54% of patients with moderately or severely increased albuminuria, the median urinary albumin to creatinine ratio (uACR) decreased from 80 to 46 mg/g. In 29% of individuals with CKD stage **≥** 3, the median uACR decreased from 66.5 to 47 mg/g at one year, and the relative risk of progression to ESKD was reduced by 70% at 2 years and by 60% at 5 years [51].

A retrospective cohort study investigated the association between bariatric surgery and the risk of mortality up to 5 years post-surgery and whether this association was modified by incident ESKD in 802 pre-dialysis CKD stage patients matched to 4933 individuals who were not undergoing surgery [52]. After adjustment for incident ESKD, bariatric surgery was associated with a 79% lower risk of mortality compared to matched controls. Furthermore, incident ESKD did not impact the association between bariatric surgery and mortality, suggesting that surgery was associated with a mortality reduction in pre-dialysis obese individuals, regardless of the development of ESKD. In a retrospective study of 149 patients undergoing either RYGB or SG, it was shown that at the 2-year follow-up, eGFR was improved in both study groups to a similar extent. Interestingly, the increase in GFR was independent of the percentage of weight loss, suggesting that other mechanisms rather than weight loss per se contribute to the improvement in renal function [53]. Moreover, in this study, progression to worse renal function following bariatric surgery correlated significantly with lower rates of hypertension and diabetes remission [53].

In a recent analysis from the Microvascular Outcomes after Metabolic Surgery (MOMS) trial, Cohen et al. assessed the hypothesis that RYGB would be more effective than the best medical treatment as a means of achieving the remission of microalbuminuria in patients with type 2 diabetes, obesity, and early-stage CKD at baseline [54]. The inclusion criteria for the study participants were uACR greater than 30 mg/g, T2D, and BMI ranging from 30 to 35 kg/m^2^. Patients had G1 to G3 and A2 to A3 CKD. Forty-nine patients were randomized to receive the best medical treatment and 51 to undergo RYGB. At the 2-year follow-up, remission from albuminuria occurred more frequently in the RYGB group (84%) compared to patients receiving the best medical treatment (56%) (risk difference 0.279; 95% CI, 39.0–70.0%). The remission of CKD, defined as the remission of albuminuria with an eGFR greater than 60 mL/min, also occurred more frequently following RYGB 81.9% (95% CI, 71.8–92.1%) compared to best medical treatment 48.2% (95% CI, 32.2–64.1%) [54].

### 2.5. Studies Using Measured GFR following Bariatric Surgery

Few prospective studies have evaluated the effect of bariatric surgery on renal function using mGFR. Chagnac et al. [18] studied eight patients with morbid obesity and nine healthy lean controls measuring inulin and PAH clearance to determine GFR and renal plasma flow, respectively. Patients with obesity had a higher GFR (145 ± 14 mL/min vs. 90 ± 5 mL/min) and higher RPF (803 ± 39 mL/min vs. 610 ± 41 mL/min) compared to the healthy volunteers. Following bariatric surgery, along with the marked weight loss, both measurements and albuminuria were significantly decreased [18]. Another study using iohexol plasma clearance to calculate the mGFR was conducted by Friedman et al. in a population of obese patients without T2D or overt nephropathy [55]. Following bariatric surgery, the unadjusted mGFR showed a significant reduction of ~17 ± 6 mL/min compared to the baseline values, suggesting the protective role of surgery-induced weight loss against hyperfiltration.

Recently, Clerte et al. [56] demonstrated that six months after bariatric surgery (RYGB or SG), the iohexol clearance rate globally increased slightly in 16 patients affected by severe obesity, with or without T2D, 25% of whom had baseline mGFR < 90 mL/min. In a subgroup analysis of seven patients displaying hyperfiltration at baseline (mGFR > 120 mL/min), the mGFR was significantly decreased and returned to normal values. Curiously, changes in body mass index (BMI) post-surgery did not correlate with the variations in mGFR [56]. This finding was confirmed in a prospective study conducted by Solini et al. [57]. Specifically, in this prospective cohort study, twenty-five obese and non-diabetic individuals showed a substantial stability in unadjusted mGFR and an improvement in adjusted mGFR, but none of the measures of adiposity at baseline were associated with ΔmGFR/BSA variations [57].

Overall, these data suggest that weight loss following bariatric surgery protects from glomerular hyperfiltration in obese patients who have excessive GFR at baseline and protects from renal failure in patients who have already experienced a decline in their GFR.

### 2.6. Meta-Analysis of Renal Function and Bariatric Surgery

Data on the effect of bariatric surgery on albuminuria and/or proteinuria were included in a 2016 meta-analysis of continuous data from 10 studies (and a total of 930 patients) and dichotomous data from 14 studies (1186 patients) [58]. After bariatric surgery, a reduced uACR and albumin excretion rates were shown. Furthermore, the risk ratio (RR) relative to baseline was reduced for both proteinuria (RR 0.31; 95% CI 0.22–0.43) and albuminuria (RR 0.42; 95% CI 0.36–0.50).

In the same meta-analysis, the impact of bariatric surgery on hyperfiltration was also assessed. Studies evaluating glomerular hyperfiltration were divided into four subgroups (those using mGFR, CrCl, adjusted eGFR, and unadjusted eGFR), and they were analyzed separately. Nine studies with continuous data (631 patients) and six studies of 514 patients with dichotomous data were included in this meta-analysis, demonstrating that the RR for hyperfiltration relative to baseline was significantly reduced after surgery (RR 0.46; 95% CI 0.26–0.82). In patients with hyperfiltration, the analysis of the available continuous data also showed significant decreases in all subgroups, although the authors drew conclusions from studies only using measured GFR (with inulin or iothalamate clearance). On the contrary, individuals with stage-2 CKD (i.e., GFR 60–90 mL/min/1.73 m^2^), for which only studies with eGFR were available, a significant increase in adjusted and unadjusted eGFR after bariatric surgery was shown. In summary, this meta-analysis showed the protective role of bariatric surgery in reducing hyperfiltration and, at the same time, in increasing GFR when renal function was already compromised [58]. The main limitation of this meta-analysis was the restricted amount of evidence due to the lack of randomized controlled trials and the differences in the timings of follow-up studies. Still, the overall heterogeneity of the outcome measures was low, suggesting a consistent response to surgery across studies [58].

The efficacy of bariatric surgery in relation to renal function and proteinuria were confirmed two years later by another meta-analysis that included 23 cohort studies, for a total of 3015 patients [59]. In fact, bariatric surgery significantly decreased proteinuria, reported in 13 of the 23 studies (mean difference—0.04 g/24h; 95% CI = −0.06 to −0.02). Reductions in albuminuria were seen after various surgical techniques, without differences for type of intervention.

GFR was assessed in 17 out of 23 studies. Two studies directly measured GFR through plasma iohexol clearance and inulin clearance, while the other studies reported 24-h creatinine clearance or Cockcroft–Gault formulas and eGFR calculated via MDRD or CKD-EPI equations.

Globally, GFR tended to be normalized across the different categories of renal impairment, being significantly reduced in the hyperfiltrating subjects and significantly improved in the CKD subgroups, 6 months or more following bariatric surgery, irrespective of the surgical method performed.

The most recent meta-analysis (2021) of six studies involving 106 patients with at least stage-3 CKD receiving bariatric surgery showed an improvement in adjusted eGFR with a mean difference of 11.64 mL/min/1.73m^2^ (95%CI: 5.84–17.45) [60]. There was no significant difference in the relative risk of having stage-3 CKD after bariatric surgery, with a relative risk of −1.13 (95%CI: −0.83 to −2.07), but there was a reduction in the relative risk of having micro and macro albuminuria (uACR > 30 mg/g) (RR = 3.03; 95%CI: −1.44 to −6.40) in three studies that included a total of 489 patients [60].

Interestingly, a meta-analysis focusing on 15 studies that reported changes in albuminuria in patients with obesity and T2D did not show any significant correlation between uACR improvement and glycemic improvement, expressed as the reduction in HbA_1c_ (r_s =_ −0.378, *p* = 0.403) or the amount of weight decrease (r_s_ = −0.306, *p* = 0.504). The researchers discussed these results and suggested that weight-independent factors such as changes in incretins following surgery-induced anatomical remodeling and/or the restoration of vascular tone and podocyte functions due to improved adiponectin level might have contributed to the reduction in albuminuria [61]. All of the above studies are summarized in Table 1.

### 2.7. Bariatric Surgery Decreases Renal Sinus Fat (RSF)

Obesity is associated with ectopic fat deposition. The accumulation of fat in the renal sinus (i.e., the perirenal hilum region at the medial border of the kidney where the ureter and the vessels enter the kidney), has been associated with higher systolic blood pressure, a higher number of antihypertensive drugs needed [62,63], a decreased GFR [62,64], and microalbuminuria [65]. From a pathophysiologic standpoint, it has been suggested that excessive fat accumulation in this specific fat depot would result in increased intra-abdominal pressure and the compression of the low-pressure renal venous structures [66,67], which would lead to the alteration of the renal hemodynamics, possibly by the activation of the renin angiotensin aldosterone system (RAAS) [67]. While several studies have shown that RSF is increased in patients with obesity, in a recent study we showed for the first time that following bariatric surgery, RSF is decreased [68]. Importantly, this decrease was associated with a remission from hypertension following bariatric surgery and a decrease in the number of antihypertensive drugs needed [68]. On the contrary, we could not detect any association with renal function, but in this study only eGFR data—rather than mGFR data—were available [68].

### 2.8. Renal Metabolism and Perfusion before and after Bariatric Surgery

Positron emission tomography (PET) represents the current gold standard for the assessment of tissue metabolic rates in humans in vivo. [^18^F]FDG is the most widely used PET tracer assessing glucose uptake rates [69], but there are several other tracers that can be used for assessing different aspects of metabolism and perfusion [70,71,72]. The various PET tracers used in the study of renal function have recently been reviewed elsewhere [72]. From a metabolic perspective, in a renal 14(R,S)-[18F]Fluoro-6-thia-heptadecanoic acid ([^18^F]FTHA) PET study, we showed that renal free fatty acid (FFA) uptake was higher in patients with obesity compared to lean individuals and that six months following bariatric surgery renal FFA uptake was still high (and not normalized) because of the ongoing catabolic state [73]. In the same study, renal volume, renal density, and renal perfusion were evaluated with computerized tomography/magnetic resonance imaging and [^15^O]-H_2_O-PET, respectively. Along with the well-known enlargement of visceral organs in obesity, patients with obesity also had a larger renal volume and decreased renal tissue density. Whereas there were no differences in renal perfusion per 100 mL of tissue volume between lean and obese individuals, total renal blood flow (thus accounting for renal volume) was larger in patients with obesity. Six-months following bariatric surgery, total renal blood flow (mL/min) and renal volume were significantly decreased, whereas renal density was increased, suggestive of the lower intrarenal accumulation of water and/or lipids [73]. The eGFR (ml/min) was also higher in patients with obesity and decreased following weight loss. Taken together, this study demonstrated via imaging that obesity leads to structural, metabolic, and hemodynamic renal changes, and that six-months following bariatric surgery these alterations (eGFR, total renal blood flow, renal volume, and renal density) are partly reversed, thereby attenuating the risk for the progression of obesity-induced chronic kidney disease.

Thus far, no study has assessed renal glucose metabolism using [^18^F]FDG-PET, but work is being carried out at the Turku PET Centre to address this aspect.

### 2.9. Bariatric Surgery and Nephrolithiasis

Nephrolithiasis occurs as a consequence of the crystallization of solutes from urine to form stones. Obesity represents a risk factor for nephrolithiasis [74], but it seems that this risk is further increased following malabsorptive bariatric surgery.

From a mechanistic standpoint, hyperoxaluria is a key element for the increased risk of nephrolithiasis. Normally, in the intestinal lumen, dietary calcium binds with oxalate to form an insoluble complex which is then excreted in the feces. This leaves a limited quantity of oxalate available for absorption. However, the malabsorption and steatorrhea that often follow extensive intestinal bypass procedures cause intraluminal calcium to bind preferentially with fatty acids, leaving larger quantities of soluble oxalate for absorption. Moreover, the colonic absorption of oxalate is also increased due to the intraluminal and mucosal alterations caused by the entry of malabsorbed fatty acids and bile salts into the colon [75]. Previous research has shown that cases of hyperoxaluria are more frequently observed in patients who have undergone RYGB or biliopancreatic diversion with a duodenal switch compared to morbidly obese patients who have not undergone surgery [76].

Apart from increased urinary oxalate, calcium oxalate supersaturation, decreased urinary citrate, and decreased total urinary volume postoperatively have also been identified as risk factors for nephrolithiasis after RYGB [77].

On the contrary, a low incidence of kidney stones has generally been observed in patients who have undergone SG or other restrictive procedures such as adjustable gastric banding [78]. A retrospective study evaluated the 24-h urine profiles of patients with obesity and a history of nephrolithiasis who underwent either RYGB or SG. The results showed that the RYGB group had significant increases in oxalate and decreases in citrate urinary excretion, while the SG group had decreased oxalate excretion and stable citrate excretion [79]. The examination of the rate of nephrolithiasis after laparoscopic RYGB versus SG showed that patients who underwent RYGB had a higher incidence of nephrolithiasis compared to those who underwent SG [80].

Importantly, a study by Semins et al. showed that following a restrictive operation (in this study gastric banding), a significantly lower incidence rate of upper urinary tract lithiasis occurred in the surgery-treated group compared to the control group of obese patients during the 2-year follow-up [81]. Thus, in patients with a significant history of nephrolithiasis, restrictive operations should be considered.

### 2.10. Acute Kidney Injury (AKI) following Bariatric Surgery

It is important to note that acute kidney injury (AKI) can ensue in the post-operative setting following bariatric surgery [82]. Risk factors for the development of AKI are a high weight, significant co-morbidities, and the use of nephrotoxic agents [82]. Additionally, following prolonged interventions, rhabdomyolysis has been described as one of the mechanisms leading to AKI, but nowadays the broad use of BS by specialized teams has substantially decreased the possible length of BS interventions to only 1–2 h, thus substantially decreasing the risk of rhabdomyolysis.

### 2.11. Future Perspectives

**Figure 1:**
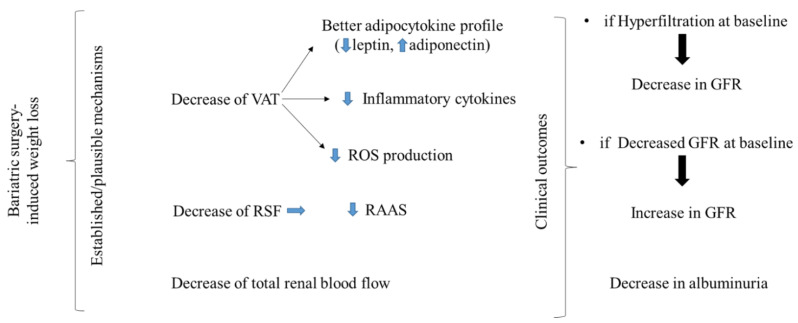
Following bariatric-surgery-induced weight loss, visceral adipose tissue deposits are decreased, as well as renal sinus fat deposits. The decrease in the former contributes to a favorable adipocytokine profile, reduction in inflammatory cytokines, and reduction in ROS production, while the latter might lead to a less-activated RAAS. Decreased total renal blood flow has also been described following bariatric-surgery-induced weight loss. Clinically, following bariatric surgery, the glomerular filtration rate is decreased in patients with glomerular hyperfiltration and increased in patients who already have a more advanced stage of chronic kidney disease. In both stages, albuminuria is decreased. These favorable outcomes ultimately decrease the rate of progression towards a more advanced stage of chronic kidney disease.

Obesity represents a significant independent risk factor for CKD, but bariatric-surgery-induced weight loss, among its other beneficial effects, can reduce glomerular hyperfiltration and restore renal function (Figure 1). It is important to underline that based on the available literature, bariatric surgery is a means of achieving significant and sustained weight loss through which improvements in renal function also occur. Whether bariatric surgery induces weight-loss-independent effects that improve renal function is currently not known, but it has been suggested by Scheurlen and colleagues based on the fact that in their meta-analysis, a change in body weight was not associated with an improvement in renal outcomes [61]. Moreover, in most studies, comparisons between restrictive and malabsorptive effects on renal function have not been conducted, probably because of small samples sizes and the belief that bariatric-surgery-induced renal outcomes are solely ascribed to weight loss. In the elegant study by Yoshino et al., the authors demonstrated that the beneficial metabolic effects of RYGB can be ascribed solely to weight loss itself (rather than to any weight-loss-independent effects) [83]. Unfortunately, in the abovementioned study, the renal outcomes following bariatric surgery and diet intervention were not assessed, and, to the best of our knowledge, no head-to-head comparison of the effects of bariatric surgery versus matched diet-induced weight loss on renal outcomes has been carried out so far. Thus, in order to answer these questions, further investigation is warranted.

In this article we also reviewed the difficulties in assessing renal function in obesity and following substantial weight loss. Even though measuring GFR is the preferred method of assessing renal function, due to its technical difficulty, this is typically only carried out in specialized centers or for research purposes. The contemporaneous assessment of albuminuria along with creatinine or cystatin-c values provides a valuable approach for large studies and scenarios wherein it is not possible to measure GFR. Recently, more economic and less invasive methods have been proposed for the measurement of GFR. These procedures involve the determination of the iohexol concentration from capillary blood samples rather than repeated blood samplings by venipuncture [84,85]. These methods could be available on a large scale in the near future, enabling the measurement of GFR in patients with morbid obesity.

## 3. Conclusions

Bariatric-surgery-induced weight loss is an effective means to ameliorate and preserve renal function in patients with obesity. The amelioration of renal and systemic inflammation; a favorable adipocytokine profile; and a reduction in the hyperfiltration state, total renal perfusion, and renal sinus fat are either established or plausible mechanisms through which bariatric-surgery-induced weight loss reverses the progression of chronic kidney disease in patients with obesity. Although an increased risk of nephrolithiasis occurs following malabsorptive interventions, and there is a risk of AKI following bariatric surgery, these complications by no means outweigh the renal benefits of bariatric surgery.

## Figures and Tables

**Table 1:** Summary of the effects of surgery-induced weight loss on GFR and albuminuria.

Reference	N	Intervention	Follow-Up	Results
*Studies using eGFR*				
Chang et al. [48]	985 patients	RYGB (96.5%), LSG (3.5%)	~4 years	BS reduced the risk of ≥30% eGFR decline and the risk of ESKD.
Shulman et al. [49]	4047 patients, of which 2010 received BS and 2037 usual obesity care	RYGB (13%), VBG (69%), GB (18%)	20 yearss	BS decreased the long-term incidence of ESRD by >70%.
Friedman et al. [50]	1449 patients (824 patients at 7 yrs)	GB, RYGB	1 and 7 years	BS resulted in lower CKD risk in a substantial proportion of patients throughout the 7-year follow-up period.
Funes et al. [51]	69 patients	LSG (42/69), RYGB (17/69)	Retrospective (16 years)	Following BS, eGFR and albuminuria were improved. The overall improvement in eGFR was greater in patients with stage-3 CKD-EPI than among those with stage-2 CKD-EPI.
Coleman et al. [52]	802 BS patients with CKD stages 3–5, vs. 4933 patients who did not undergo BS	RYGB, sleeve gastrectomy, GB	Retrospective (5 years)	BS was associated with a 79% lower 5-year risk of mortality compared to matched controls.
Holcomb et al. [53]	149 patients	RYGB, LSG	2 years	eGFR was improved similarly in both groups.
Cohen et al. [54]	51 RYGB, 49 OMT	RYGB vs. OMT	2 years	RYGB led to remission from CKD in 81.9% and remission from albuminuria in 84%.
*Studies using mGFR*				
Chagnac et al. [18]	8 patients and 9 controls	gastroplasty	12 months	Patients with obesity had hyperfiltration and increased RPF flow at baseline. After surgery, GFR and RPF were both decreased.
Friedman et al. [55]	36 patients	type of surgery not mentioned	~10 months	Reduction in hyperfiltration following bariatric surgery.
Clerte et al. [56]	16 patients	RYGB or SG	6 months	mGFR increased in patients who had reduced GFR at baseline, and decreased in patients who had hyperfiltration at baseline.
Solini et al. [57]	25 non-diabetic patients	RYGB	1 years	mGFR remained stable; mGFR/BSA was increased.

BS: bariatric surgery; e/mGFR: estimated/measured glomerular filtration rate; GB: gastric banding, OMT: optimal medical treatment; RPF: renal plasma flow; RYGB: Roux-en-Y gastric bypass; LSG: laparoscopic sleeve gastrectomy; ESKD: end-stage kidney disease; VBG: vertical banded gastroplasty; CKD: chronic kidney disease; CKD-EPI: chronic kidney disease epidemiology collaboration; BSA: body surface area.
